# UHPLC-MS-Based Metabolomics of Goat Bone Waste and the Antioxidant, Hemocompatibility, and Fibrinolytic Potential of Its Aqueous Extract

**DOI:** 10.3390/ijms27219953

**Published:** 2026-11-06

**Authors:** Ahmed Hamad Alanazi, Hallouma Bilel, Aml M. Asran, Sherifa H. Ahmed, Amnah Salem Al Zbedy, Amr Mohammad Nassar

**Affiliations:** 1 Chemistry Department, College of Science, Jouf University, Sakaka 72341, Saudi Arabia; ahanazi@ju.edu.sa (A.H.A.); hbilel@ju.edu.sa (H.B.); ahasanen@ju.edu.sa (A.M.A.); 2 Biology Department, College of Science, Jouf University, Sakaka 72341, Saudi Arabia; shhamohmoud@ju.edu.sa; 3 Department of Chemistry, Al-Qunfudah University College, Umm Al-Qura University, P.O. Box 1109, Al-Qunfudah 21912, Saudi Arabia; asazubidi@uqu.edu.sa

**Keywords:** HPLC, bone, waste, extract, medical applications

## Abstract

Recycling of food waste and animal by-products in order to produce valuable bioorganic products has been playing an important role in the green chemistry field. In this study, the chemical composition of goat bone wastes was analyzed through untargeted Ultra-High-Performance Liquid Chromatography-Quadrupole-Orbitrap Mass Spectrometry (UHPLC-Q-Orbitrap MS) metabolomics in dual electrospray ionization (ESI) modes. Using accurate-mass tandem mass spectrometry (MS/MS) database searching, it was possible to annotate various compounds present in the bone waste samples, such as collagen-derived amino acids, peptides, organic acids, nucleotides, and lipids. In parallel, we also investigated the biological activity of the aqueous extract of goat bone matrix (GBAE). The GBAE showed potent antioxidant activity since it was capable of completely neutralizing (100%) DPPH radicals at a concentration of 100 mg/mL. Moreover, the hemolytic activity of GBAE was studied and found to be very low (<1% with human erythrocytes), indicating its weak in vitro hemotoxicity. When oxidative stress was generated via lead acetate (0.625 ppm), GBAE provided a considerable protective effect, leading to 65.0%, 67.5%, and 73.7% hemolysis reduction at concentrations of 200, 400, and 800 mg/mL, respectively. Additionally, preliminary in vitro findings suggested that GBAE exhibited fibrinolytic potential and anticoagulant activities. GBAE showed dose-dependent thrombolytic and anticoagulant activity, with a significant increase in the percentage of clot lysis and coagulation time up to 36 min, compared with 13 min in the negative control.

## 1. Introduction

The sustainable utilization of food waste and animal byproducts offers possibilities for the exploration of novel biomaterials [1]. Goat bones offer an abundant and affordable source of type I collagen, non-collagenous proteins, lipids, and biogenic minerals [2]. While traditional bone processing typically involves heating of the bone to form hydroxyapatite [3], chemical analysis could provide further insight into the composition of this complex material [4].

Untargeted ultra-high-performance liquid chromatography with quadrupole-orbitrap tandem mass spectrometry (UHPLC-Q-Orbitrap MS) can help analyze a wide variety of chemicals [5,6]. The two complementary ESI modes can help expand the range of possible ion detection: positive ESI tends to work well for compounds with nitrogen atoms and zwitterions, while negative ESI favors acids and deprotonated molecules [7,8,9]. Modified biphasic extraction approaches, such as Bligh–Dyer-type extractions, may be employed for the extraction of polar and lipophilic fractions [10]. Concurrently, aqueous extraction affords another method of preparing samples for studying the bioactivities of water-soluble constituents in biological assays. Indeed, aqueous extraction of the bone matrix may yield peptides and amino acids including glycine, proline, and hydroxyproline as well as phospholipids and sphingolipids [11,12]; these constituents have reportedly provided electron donors, chelators of trace elements, and increased cellular resistance to oxidative stress [13,14]. As solvent choice and processing parameters affect the compounds extracted, the metabolomic analysis performed on the material following biphasic extraction cannot be considered the same as the composition of an aqueous extract prepared independently.

By-products from cattle, pigs, fish, and poultry bones have been mainly utilized for the production of hydroxyapatite, gelatin, or collagen hydrolysates via calcination or chemical/enzymatic hydrolysis [15,16,17], and antioxidant peptides have been isolated from enzymatic hydrolysates of bone collagen [18]. Unbiased LC-MS-based approaches for studying the metabolome of bone tissue have only become possible relatively recently, being mostly applicable to human cortical bone in forensics [19]. By comparison, the small-molecule composition of goat bone has rarely been studied despite the significant amount of goat bone produced in areas where goat meat is extensively consumed. During the course of our literature review, we could find no earlier reports on the combination of dual-polarity untargeted LC-MS metabolomics of goat bone with the in vitro testing of the antioxidant, erythrocyte-related and clot-related properties of solvent-free aqueous extract. Thus, this research aims to develop an inclusive approach towards valorization of goat bone waste. Through the use of the two-polarity UHPLC-Q-Orbitrap MS technique, we have identified, classified and characterized the complex metabolomics of goat bone waste. In addition, the biological activity of its derived goat bone aqueous extract was systematically evaluated, highlighting its strong antioxidant capacity, compatibility with human erythrocytes, protection against oxidative hemolysis caused by lead acetate and its thrombolytic and anticoagulant activity.

## 2. Results and Discussion

### 2.1. Chromatographic Profiling and Untargeted Metabolomic Analysis

**Figure 1:**
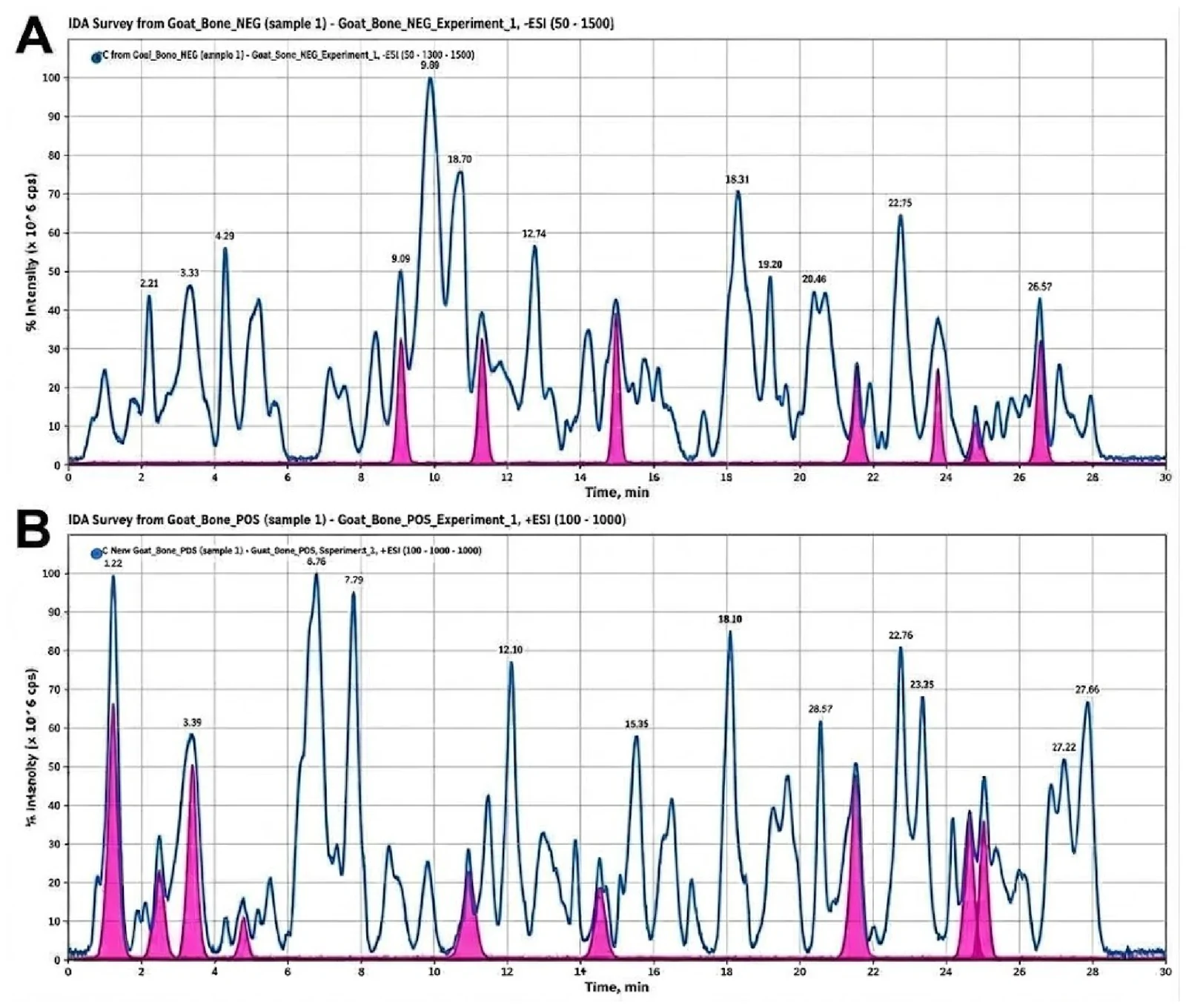
Total ion chromatograms (TICs) of goat bone acquired by UHPLC–MS under dual ionization modes: (**A**) negative ESI mode and (**B**) positive ESI mode. Blue lines denote total ion intensities, while pink shaded peaks represent the target integrated metabolites.

A complete metabolic map of goat bone samples was attained via an untargeted liquid chromatography-mass spectrometry analysis using two electrospray ionization modes [20,21]. Throughout the analysis period, it was evident that the total ion current chromatograms generated in negative and positive modes displayed an extraordinary level of complexity, as shown in Figure 1. The early-eluting peaks contained predominantly highly polar compounds, such as low molecular weight amino acids, organic acids, and phosphorylated compounds, whereas the late-eluting peaks displayed a high abundance of non-polar compounds like fatty acids, bile acids, and lipids [22].

**Figure 2:**
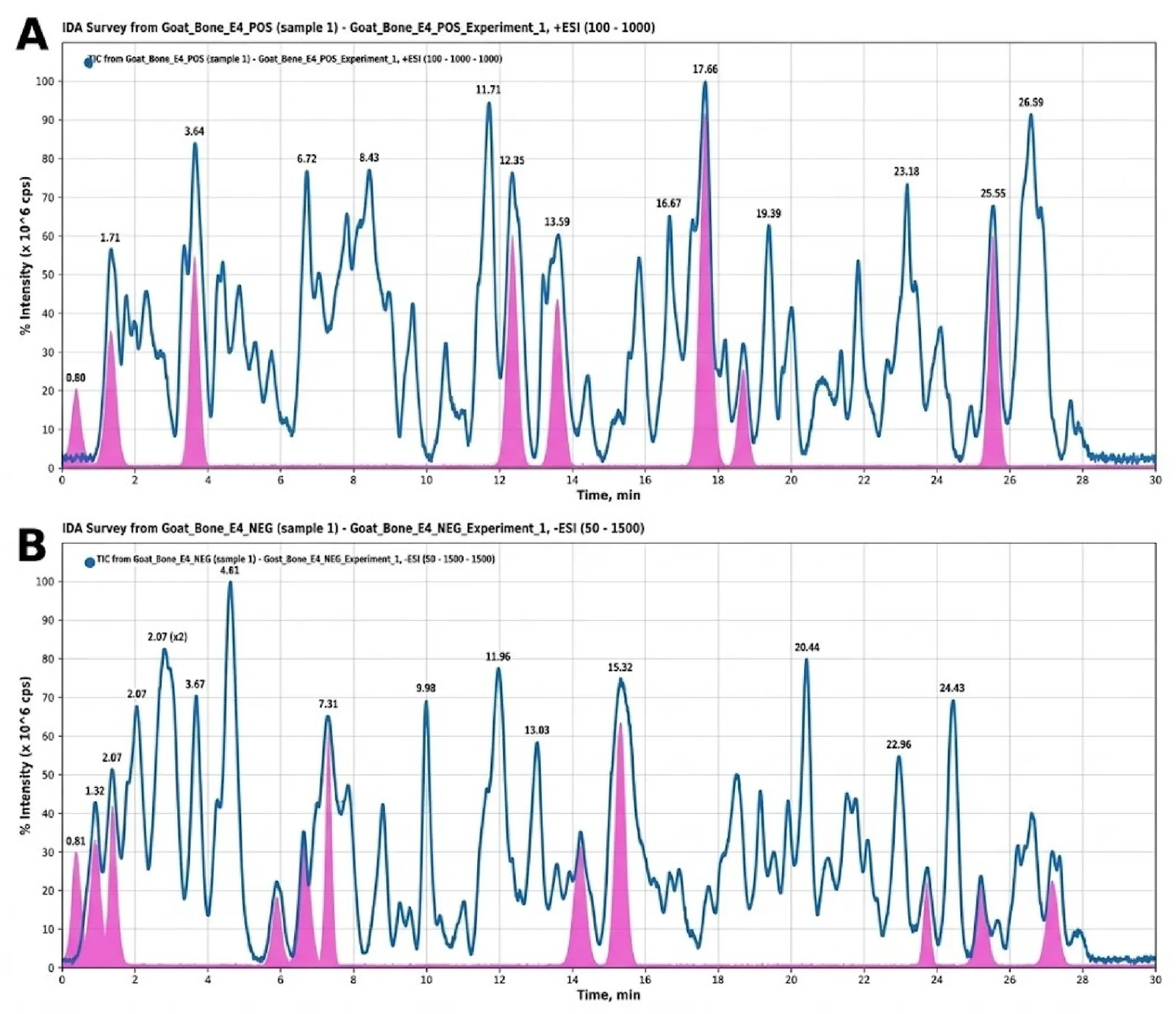
Total ion chromatograms (TICs) of goat bone extracts acquired in negative (**A**) and positive (**B**) ESI modes, demonstrating consistent retention-time profiles between the primary sample and the biological sample. Blue lines denote total ion intensities, while pink shaded peaks represent the target integrated metabolites.

In order to validate the analytical procedure’s robustness and account for biological variability that occurs naturally, an independent biological replicate of the sample analyzed in a similar manner was studied alongside it [23,24]. The structural similarities of the two samples were observed by comparing the primary sample and its replicate side by side, and the results obtained showed remarkable structural similarity with respect to their total ion chromatograms, with excellent stability in terms of performance, peak density and repeatable retention times in the early, middle and late elution phases, as shown in Figure 2. While some variations in peak intensities could be observed in the two samples, these differences are purely biological in nature with no significant effect on any analytical factors.

**Figure 3:**
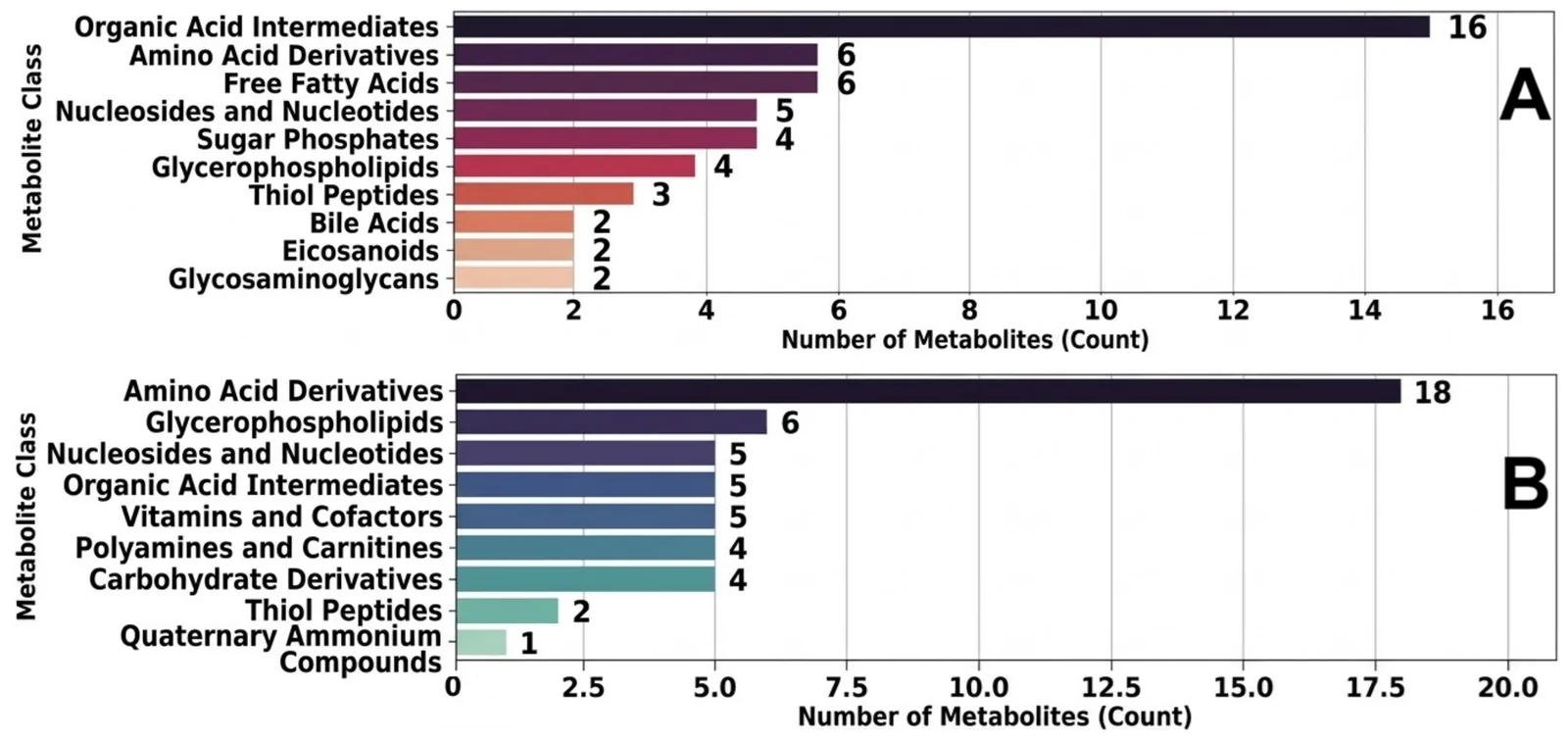
Comparative metabolite class distribution in goat bone extracts analyzed in negative (**A**) and positive (**B**) ionization modes.

The use of both electrospray ionization methods turned out to be crucial for obtaining complete chemical coverage, resulting in different yet highly complementary profiles within each category of annotated metabolites, as shown in Figure 3. Negative electrospray ionization was used to identify acidic and phosphorylated metabolites, free fatty acids, and lipid metabolites, while positive electrospray ionization selectively identified nitrogen-containing and zwitterionic metabolites, such as collagen peptides, amino acids, nucleotides, and choline-containing metabolites [25,26]. From a biochemical point of view, such metabolic diversity directly reflects the dual nature of bone: the presence of amino acids and peptides reveals the fact that type I collagen peptides and structural proteins were successfully recovered, while the identification of choline-containing metabolites, nucleotides, and lipids reveals the presence of marrow constituents and membrane metabolites [27].

The relative quantitative metabolomic profiles of goat bone extracts obtained in negative ([M − H]^−^) and positive ([M + H]^+^) ionization modes are presented in Table 1 and Table 2, respectively, with the top 50 most abundant metabolites in each mode.

### 2.2. Antioxidant Activity of GBAE

**Figure 4:**
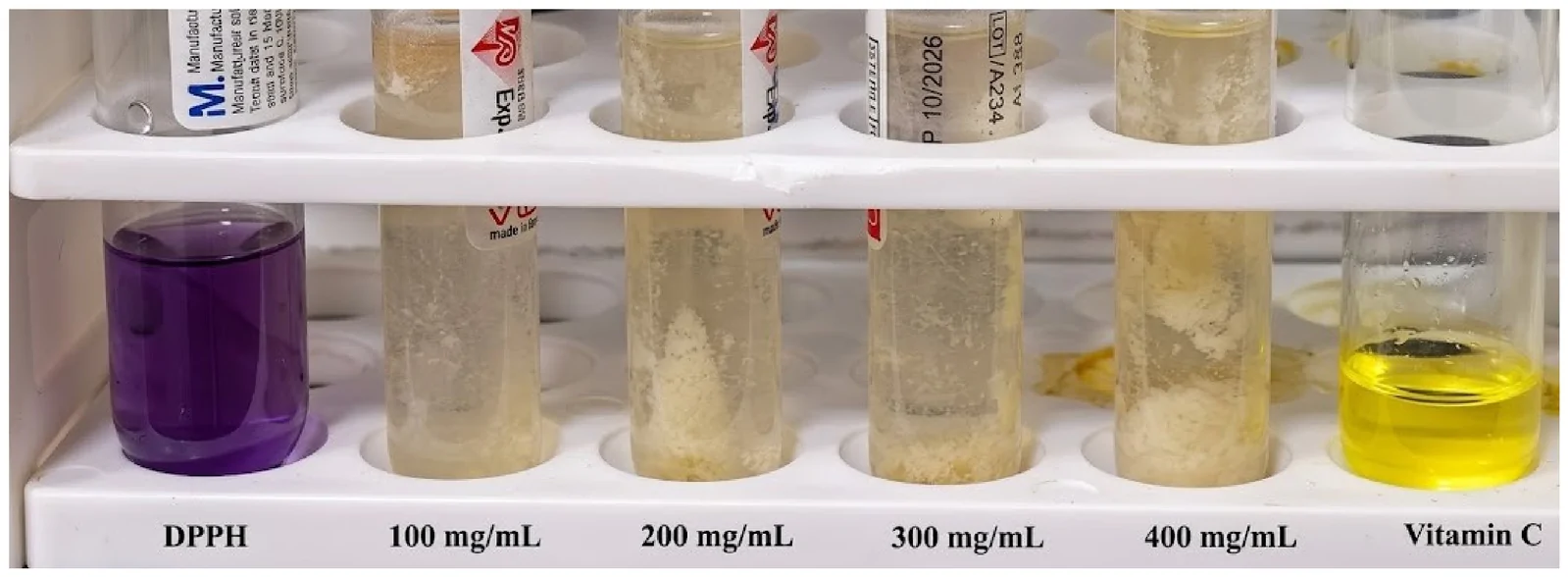
Antioxidant activity of GBAE at different concentrations.

In addition to the detailed chemical composition analysis, testing the biological activity showed that GBAE exhibited extremely high levels of antioxidant activity, as shown in Figure 4. Using the method of 2,2-diphenyl-1-picrylhydrazyl scavenging assay [28], and aided by additional UV-Vis spectroscopy analysis, the aqueous extract proved to possess extremely high ability to neutralize stable free radicals [29]. It is particularly worth noting that the aqueous extract managed to completely neutralize all the radicals (100%) at a concentration as low as 100 mg/mL.

**Figure 5:**
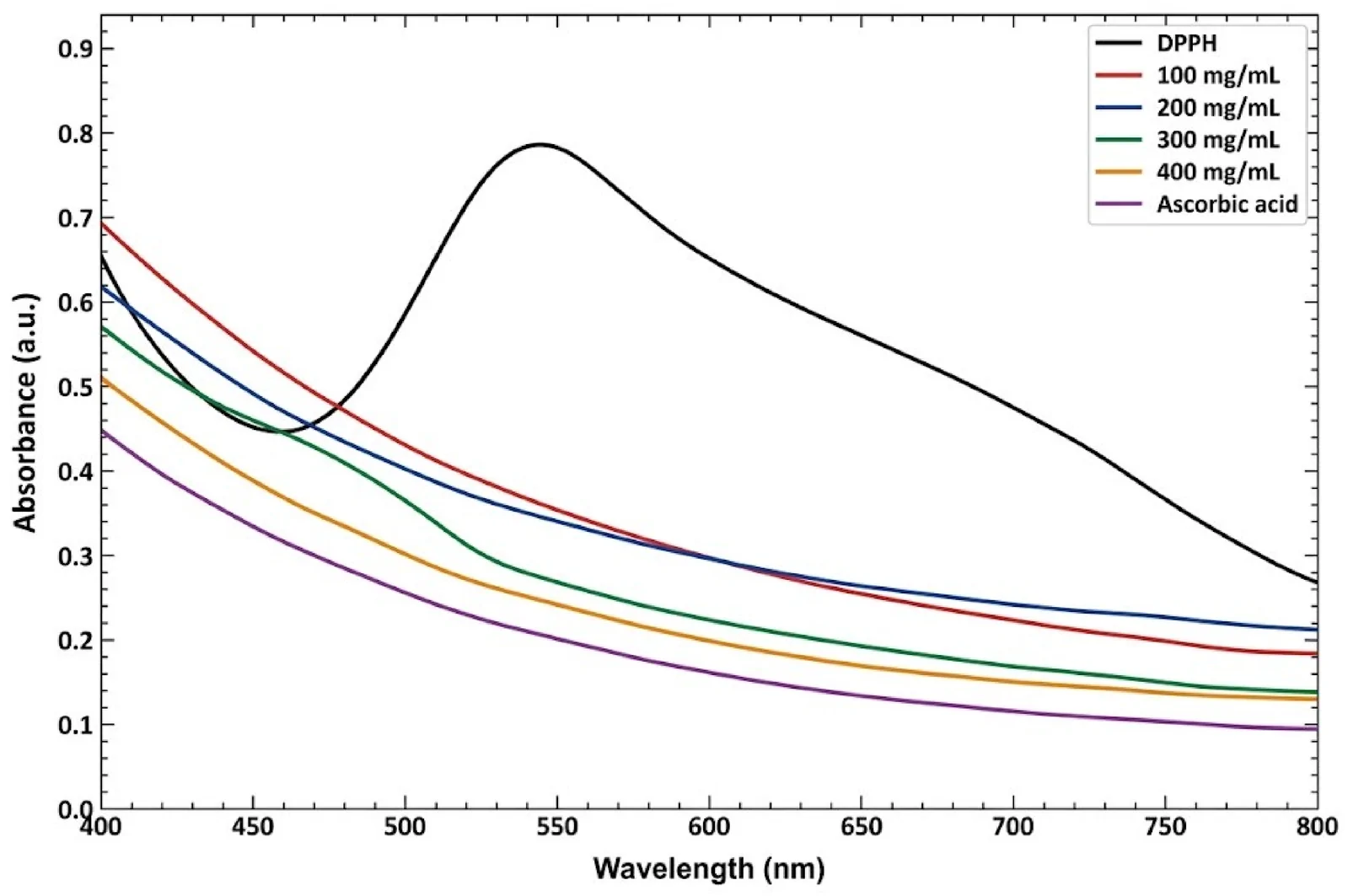
Visible absorption spectra of the DPPH free radical in the absence (control) and presence of the aqueous extract at different concentrations (100–400 mg/mL) and ascorbic acid standard over the wavelength range of 400–785 nm.

The complementary visible absorption spectroscopy provides additional support to the aforementioned results by showing a significant decrease in absorbance at 517 nm [30], with increasing GBAE concentration from 100 mg/mL to 400 mg/mL as shown in Figure 5. This notable radical scavenging capacity is due to the high content of water-soluble biological molecules contained in the goat bone extracts, which include low molecular weight collagen peptides and free amino acids [31]. Structural bone proteins (mainly Type 1 Collagen) produce many peptides after extraction, which contain amino acids like glycine, proline and hydroxyproline [32]. Biologically active components act as hydrogen atom and electron donors and are able to donate electrons or hydrogen atoms to neutralize nitrogen-based free radicals and provide resistance to oxidative stress [33,34]. Therefore, these results suggest that goat bone extracts contain natural antioxidants with great application potential.

### 2.3. Hemocompatibility and Antihemolytic Protective Efficacy Against Heavy Metal-Induced Oxidative Stress

**Figure 6:**
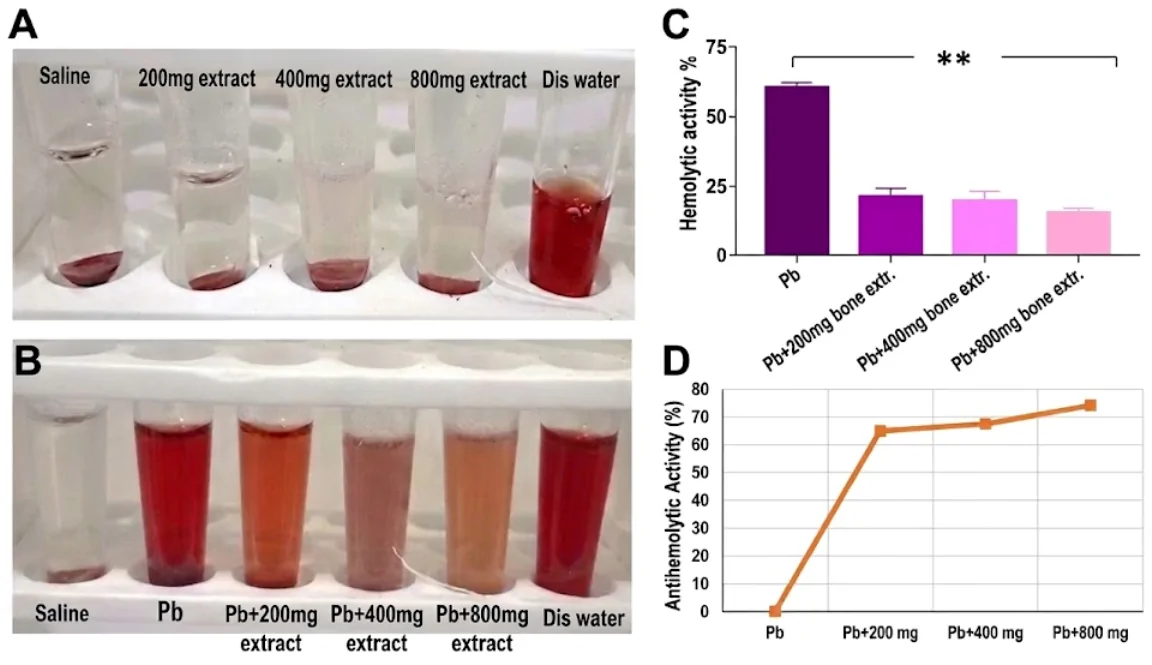
Hemocompatibility of aqueous goat bone extracts (200, 400, and 800 mg/mL) on human red blood cells (**A**). Macroscopic visualization of antihemolytic protection against lead acetate (0.625 ppm)-induced toxicity (**B**). Quantitative analysis of hemolytic activity percentage (** *p* ≤ 0.001) versus positive control (**C**). Percentage of change of hemolytic activity of GBAE against lead acetate induced hemolysis (**D**).

Hemocompatibility testing should be strictly conducted for any biologically derived substance or biomaterials to be used in biomedical applications [35,36]. It was observed through in vitro tests that the GBAE showed excellent biosafety with insignificant hemolytic activity less than 1% at concentration levels from 200 to 800 mg/mL compared with the control groups (Figure 6A). The negligible effect on human red blood cells highlights the high safety and blood compatibility of the material [37]. The effectiveness of the extract in protecting red blood cells from excessive oxidative stress caused by lead acetate (0.625 ppm) was thoroughly studied [38,39]. The presence of this metal leads to increased membrane instability, causing significant hemolysis in erythrocytes, which reaches up to 60% compared to the negative control (*p* ≤ 0.001) (Figure 6B). Nevertheless, the preliminary treatment with GBAE showed a significant, concentration-dependent response to protect red blood cells against hemolysis induced by lead acetate (*p* ≤ 0.001). Indeed, the percentage change in hemolytic activity was decreased by 65.0%, 67.5%, and 73.7% for extract concentrations of 200, 400, and 800 mg/mL, correspondingly (Figure 6C,D). These dynamic defensive mechanisms clearly correlate with the powerful antioxidant activity and unique biochemical profile of the extract [40]. The presence of particular amino acids, including glycine, cysteine, and glutamine, acts as an important source of intracellular precursors of glutathione formation, providing additional defenses against reactive oxygen species and stabilizing the structure of the lipid bilayer [41]. Additionally, lipids and other lipid-based compounds detected in the metabolomic analysis, such as ceramides and sphingomyelins, which have the backbone structure of sphingosine, are extremely important in maintaining membrane fluidity, cell survival signaling, and mitochondrial protection from oxidative stress [42]. These dynamics correspond to the recent development of goat bone-derived biomaterials as a biologically based treatment modality against heavy metal toxicity [31].

### 2.4. Fibrinolytic and Anticoagulant Bioactivities of GBAE

**Figure 7:**
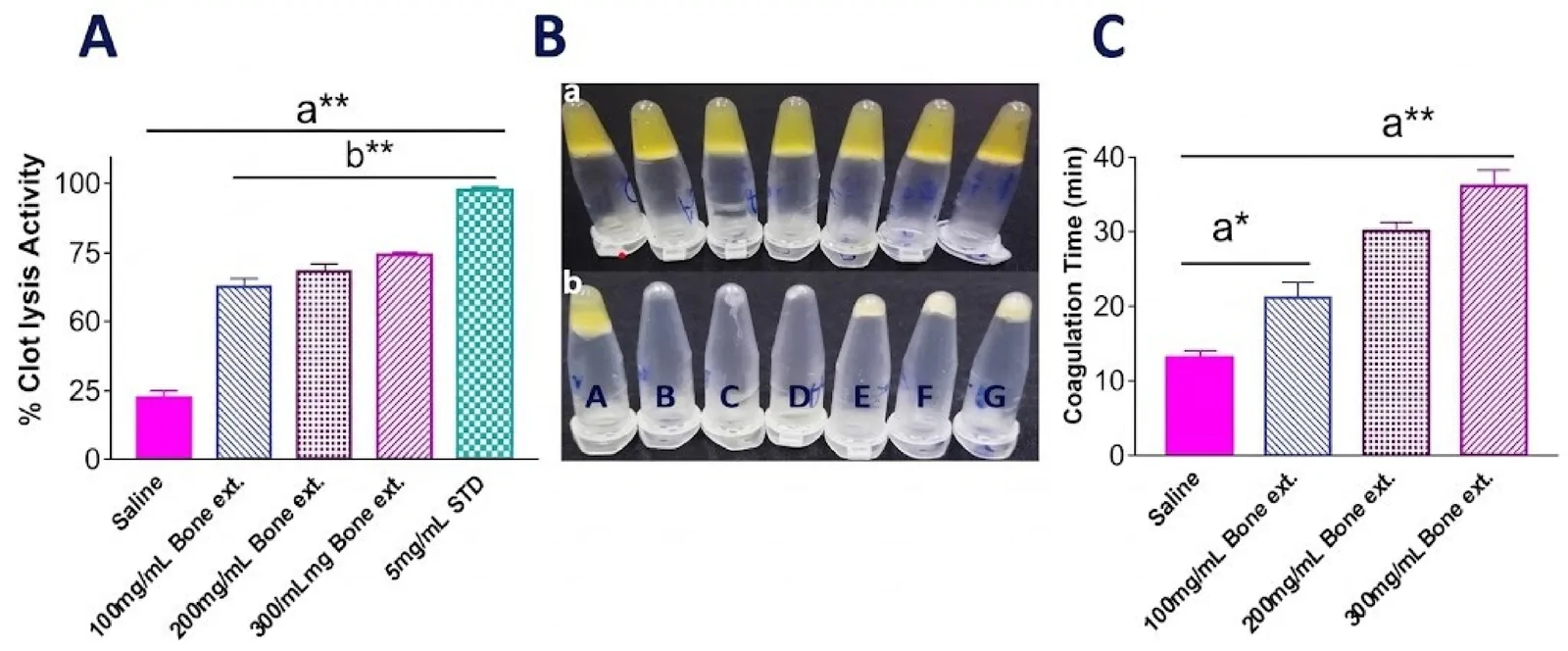
(**A**) In vitro fibrinolytic activity of (100, 200, 300 mg/mL) bone extract and (5 mg/mL) STD Actilyse. (**B**) A representative image of clot lysis activity: a—Before treatment, b—After treatment (A—negative control; B, C, D (5, 2.5, 1.25 mg/mL) Actilyse; E, F, G (300, 200, 100 mg/mL) bone extract, respectively). (**C**) In vitro anticoagulation activity of (100, 200, 300 mg/mL) bone extract. a* *p* ≤ 0.05, a** *p* ≤ 0.001 versus negative control, and b** *p* ≤ 0.001 versus positive control.

The efficiency of GBAE to modulate hemostasis was investigated in light of its antioxidant and antihemolytic activities. In vitro clot lysis analysis has shown impressive, dose-dependent ability in the dissolution of blood clots in humans with effective dissolution rates comparable to biochemical standards (Figure 7A,B). Numerically, clot lysis ability significantly increased from 22% in the saline control group to 62%, 68%, and 74% at extract concentrations of 100, 200, and 300 mg/mL, respectively (*p* ≤ 0.001) compared to negative and positive controls. At the same time, an efficiency of 97% was demonstrated by the reference drug Actilyse (plasminogen activator) [43]. The strong fibrinolytic activity is mainly attributed to the synergistic effect of active proteolytic fragments and protein hydrolysates, which are capable of effectively binding and dissolving the cross-linked fibrin network [44]. Consistent with Furlan et al. [45], bone extract’s fibrinolytic action may be associated with endogenous fibrinolytic enzymes such as matrix metalloproteinases (MMPs), tissue-type plasminogen activator (tPA), urokinase-type plasminogen activator (uPA), and plasminogen. Fibrin elimination, fracture healing, and extracellular matrix remodeling are all facilitated by these elements. It was known that the prokinase receptor (uPAR) glycoprotein and its ligand, urokinase uPA are expressed by both osteoblasts and osteoclasts. The anticoagulant analysis through plasma coagulation time has shown a regulatory effect on both the intrinsic and extrinsic coagulation cascades. Figure 7C showed a substantial, dose-dependent prolongation of plasma coagulation times ranging from 13 min in the negative control group to 21, 30, and 36 min at 100, 200, and 300 mg/mL concentrations, respectively (*p* ≤ 0.05, 0.001) compared to the negative control. It was reported that the STD Inhixa drug coagulation time lasted < 24 h. Instead of causing a severe risk of hemolysis and spontaneous lysis, the extract caused a moderate increase in clotting time, suggesting an effective and stable interaction between the extract and main coagulation factors. Furthermore, bioactive compounds with important anticoagulant capabilities have been found in bone extract. Glycosaminoglycans, including chondroitin sulfate and dermatan sulfate, together with certain bioactive peptides, are the main constituents in anticoagulant function. Glycosaminoglycans from bone extract act as indirect inhibitors of thrombin (Factor IIa). They enhance the activity of Antithrombin III (ATIII) or Heparin Cofactor II (HCII), which are natural inhibitors of the coagulation cascade [46]. Mechanically, the occurrence of specific amino acids and peptide-mineral complexes in the matrix allows the formation of electrostatic interactions with the main coagulation enzymes, resulting in thrombin inhibition and reduced platelet aggregation [47]. Overall, these multifunctional hemostatic properties make the biogenic GBAE good candidates for biomedical and cardiovascular applications.

## 3. Materials and Methods

### 3.1. Materials

Fresh goat femoral bone samples (10 g) were collected from a local market in Sakaka, Al-Jouf, Saudi Arabia. Analytical-grade solvents, including LC-MS grade methanol, acetonitrile, formic acid, ammonium acetate, ammonium hydroxide, isopropanol, EDTA, and butylated hydroxytoluene (BHT), were purchased from Sigma-Aldrich (St. Louis, MO, USA) and Fisher Scientific (Loughborough, UK). High-purity chloroform (HPLC grade) designated for lipid extraction, along with other auxiliary solvents (≥99% purity), was acquired from the same commercial vendors, passed through 0.2 um syringe filters, and degassed before use. Ultrapure water was freshly generated using a Milli-Q purification system. The chemicals required for the subsequent biological activity, including 2,2-diphenyl-1-picrylhydrazyl (DPPH), ascorbic acid, lead acetate, calcium chloride, and phosphate-buffered saline, were purchased from Sigma-Aldrich (St. Louis, MO, USA). The reference clot lysis (Actilyse, 50 mg) and the anticoagulant reference (Inhixa, 40 mg) were obtained from standard clinical suppliers.

### 3.2. Instruments

Chromatographic separation and high-resolution mass spectrometric profiling were conducted on an ultra-high-performance liquid chromatography system coupled with a quadrupole-Orbitrap mass spectrometer (Thermo Fisher Scientific, Waltham, MA, USA). Sample preparation, phase separation, and processing steps utilized a refrigerated high-speed centrifuge (Eppendorf AG, Hamburg, Germany). Spectrophotometric and colorimetric analyses for the antioxidant evaluations were recorded on a UV–Vis spectrophotometer (Shimadzu Corporation, Kyoto, Japan). Temperature-controlled incubations, water baths, and biological assays were conducted in well-precisioned equipment supplied by Memmert GmbH + Co. KG (Schwabach, Germany).

### 3.3. Preparation of Goat Bone Aqueous Extract (GBAE)

The goat bone sample was washed with Milli-Q water to remove any leftover soft tissues and contaminants. Then the bones were air-dried and ground for subsequent aqueous extraction. These cleaned and dried bones were then crushed and powdered using mechanical means. In order to prepare GBAE, the bone powder was boiled in Milli-Q water for 2 h, during which time it was stirred. Filtration was done using filter paper, after which the clear GBAE was collected and preserved to be used as the aqueous extract for analysis and biological tests.

### 3.4. Chromatography Experiment (UHPLC–MS Analysis)

The goat femoral bones (epiphysis and diaphysis) were snap-frozen in liquid nitrogen and kept at −80 °C until further processing. The frozen bone samples were milled into powder with particle size <100 μm. To achieve partial demineralization, 500 mg of the bone powder was incubated for 2 h at 4 °C in 5 mL of 0.5 M EDTA buffer (pH 7.4) with gentle agitation and washed three times with PBS buffer (pH 7.4). Afterward, the samples were subjected to freezing and lyophilization for 24 h. The dried samples were aliquoted into 200 mg portions and kept at −80 °C until further processing. Metabolites were extracted using a modified Bligh–Dyer biphasic method, which is used for simultaneous recovery of polar and lipophilic metabolites. Lyophilized bone powder (200 mg) was extracted with methanol: chloroform: water (2:2:1.8, *v*/*v*/*v*) at 10 μL mg^−1^ of the tissue (2 mL total volume). The extraction solvent contained 50 μM BHT, 1 mM EDTA, and 10 mM sodium fluoride to prevent oxidative and enzymatic degradation. Samples were homogenized using 2.8 mm ceramic beads (6500 rpm, 2 × 30 s with cooling intervals), vortexed, and incubated at 4 °C for 15 min. Phase separation was accomplished via centrifugation (16,000× *g*, 10 min, 4 °C). The upper aqueous-methanol fraction (polar compounds) and lower chloroform fraction (lipids) were separated and dried under reduced pressure at 45 °C. Polar fractions were resuspended in acetonitrile: water (50:50, *v*/*v*) with 0.1% formic acid. Lipid fractions were resuspended in acetonitrile: water (90:10, *v*/*v*) with 5 mM ammonium acetate. Samples were centrifuged and loaded into autosampler vials for LC–MS analysis. Sample E4 was treated in the same way as a biological replicate. A chromatographic separation of the samples was carried out in both positive and negative electrospray ionization (ESI) modes in the binary gradient mode. Mobile Phase A in positive ion mode was composed of 0.1% formic acid in water, whereas Mobile Phase B was composed of 0.1% formic acid in acetonitrile (pH 2.7–3.0). Mobile Phase A in negative ion mode was composed of 5 mM ammonium acetate in water (pH 7.0 ± 0.2), and Mobile Phase B was composed of acetonitrile. The gradient was set as follows: 95%A, 5%B isocratic for 2 min, 50%B linear gradient up to 10 min, 95%B up to 15 min, isocratic hold till 18 min, followed by return to initial conditions for 20 min with a constant flow rate of 0.30 mL min^−1^. All mobile phases were prepared and filtered before use.

### 3.5. Antioxidant Activity of GBAE (DPPH Radical Scavenging Assay)

In evaluating the antioxidant capacity of GBAE, the standard method for 2,2-diphenyl-1-picrylhydrazyl (DPPH) radical scavenging activity was used. It is based on the ability of antioxidant components to reduce the stable nitrogen-centered radical compound by donating hydrogen atoms or electrons. Upon reduction, the dark purple color of the DPPH solution changes into pale yellow, which can be measured via a reduction in absorbance at a wavelength of 517 nm. A fresh 0.1 mM DPPH solution was prepared in acetone-water solution (1:4, *v*/*v*). 2 mL of the solution was added to 1 mL of the GBAE at different test concentrations (100, 200, 300, and 400 mg/mL) along with the ascorbic acid used as a reference antioxidant standard. Reaction mixtures were kept under dark conditions for 30 min to ensure completion of the reaction. For each GBAE concentration, a corresponding sample blank containing 1 mL of GBAE and 2 mL of solvent (acetone- water solution (1:4, *v*/*v*)), without DPPH, was prepared. The absorbance of each sample was corrected using its corresponding blank through the spectrophotometer’s blank-correction function to account for the intrinsic absorbance of the extract matrix. The radical scavenging activity was estimated as a percentage using the formula presented in Equation (1) [48]:

(1) $$\mathrm{DPPH scavenging activity}\left(\%\right)=\frac{A_{\mathrm{control}}-A_{\mathrm{sample}}}{A_{\mathrm{control}}}\times 100$$

where *A*_control_ is the absorbance of the DPPH control solution (DPPH + solvent), and *A*_sample_ is the net corrected absorbance of the extract reaction mixture. All measurements were conducted in triplicate, and data are expressed as the mean ± standard deviation (SD).

### 3.6. Hemolytic and Antihemolytic Activities of GBAE

For the in vitro evaluation of hemocompatibility and biosafety of GBAE, the hemolytic property of the extract was determined using fresh human RBCs. donated by three volunteers. 100 µL/mL of the extract at concentrations of 200, 400, and 800 mg/mL was mixed with 100 µL/mL of erythrocytes and 2 mL of physiological saline. The antihemolytic ability of the extract against the heavy metal lead acetate solution to induce oxidative hemolysis was assessed. Firstly, the basal hemolytic activity of lead acetate was determined by treating 100 µL of lead acetate (0.625 ppm) with 100 µL of erythrocytes. In the antihemolytic test, erythrocytes were pre-incubated with 100 µL/mL of the extract (at concentrations of 200, 400, and 800 mg/mL) for 1 h, followed by the addition of 100 µL of lead acetate (0.625 ppm). All reactions were incubated at 37 °C for 24 h and later centrifuged at 2500 rpm for 10 min. The absorbance of the supernatant solutions was determined by spectrophotometry at a wavelength of 540 nm. Normal saline and distilled water were used as the lowest (0%) negative control and highest (100%) positive control values, respectively, with four different replicates for each test system. The percent hemolysis was then calculated based on Equation (2) [49]:

(2) $$\mathrm{Hemolytic activity}\left(\%\right)=\frac{A_{\mathrm{sample}}-A_{\mathrm{saline}}}{A_{\mathrm{distilled water}}}\times 100$$

where A_sample_ is the absorbance of the test extract, A_saline_ is the absorbance of the negative control, and A_distilled water_ is the absorbance of the positive control.

### 3.7. Fibrinolytic and Anticoagulation Activities of GBAE

Fibrinolytic activity in vitro of the extract was assessed by adopting a method described by Prasad et al. [43]. Specifically, pre-weighed sterile 2 mL Eppendorf tubes were prepared by mixing 0.25 mL of plasma with 50 µL of 1% calcium chloride. The mixture was shaken well and allowed to stand at 37 °C for 45 min to permit blood clot formation. Weighing of clots was done by subtracting the original empty weight of the tube from its weight after formation of the clot. Thereafter, 200 µL of goat bone extract at varying concentrations (100, 200 and 300 mg/mL), 200 µL of (5, 2.5, 1.25 mg/mL) Standard reference positive control Actilyse (Alteplase, 50 mg) or normal saline (negative control) was added to the previously formed clot. Incubation of the tubes at 37 °C for 24 h followed. Afterward, the resultant fluid after clot lysis was carefully withdrawn, and the tubes were weighed to determine the weight difference. Four different test replicates were done. Percentage clot lysis was estimated using Equation (3) [50]:

(3) $$\mathrm{Clot lysis}\;\%=\frac{\mathrm{Clot weight before lysis}-\mathrm{Clot weight after lysis}}{\mathrm{Clot weight before lysis}}\times 100$$

Likewise, the in vitro anticoagulation action of the extracts was studied using modified methods that are similar to Oyaizu et al. [51] and Chegu et al. [52]. Individual reaction vessels were made by mixing 200 µL of the extract solution (at 100, 200, and 300 mg/mL), the Inhixa standard drug (40 mg), or saline with 0.25 mL of plasma and 50 µL of CaCl_2_ solution. The reaction mixture was incubated in the water bath at 37 °C, and the coagulation time was recorded with a stopwatch. Three duplicates of each experiment were performed. Statistical processing of the obtained data on aqueous goat bone extract was performed in the GraphPad Prism software, version 3, using the *t*-test, *p* ≤ 0.05. Results were presented as mean ± standard Error (SE).

## 4. Conclusions

A thorough biochemical analysis of the aqueous extract from goat bones was performed using metabolomics via UHPLC-MS analysis. Bio-waste valorization was coupled with possible biomedical applications via metabolomics analysis. Biochemical analysis of the aqueous extract showed the presence of nucleosides, organic acids, bile acids, carbohydrates, phosphates, glycerophospholipids, and lipids. In preliminary in vitro experiments, the aqueous extract demonstrated remarkable antioxidant activity (100% inhibition at 100 µg/mL), as well as excellent hemocompatibility (<1% of RBC lysis). Moreover, the aqueous extract showed antihemolytic protective activity against lead acetate-induced oxidative stress. In addition, the aqueous extract possesses clot lysis activity in a dose-dependent manner (up to 74%) and extended plasma coagulation time (up to 36 min). Nevertheless, the presented results were obtained only in preliminary in vitro experiments. Due to the complexity of the biochemical composition of the extract, any connections between the particular metabolite and its effect should be considered with caution. Thus, future in vivo experiments are required to validate the potential applications of goat bone aqueous extract as a sustainable bioactive formulation for therapeutic and biomedical interventions.

## Figures and Tables

**Table 1:** Top 50 abundant annotated metabolites in goat bone extract under negative ionization mode ([M − H]^−^).

Annotated Metabolite	RT (Min)	Experimental *m*/*z*
Citric acid	2.27	191.0196
Acetoacetic acid	1.56	101.0239
Glycocholic acid	7.38	464.3010
Fructose 1,6-bisphosphate	7.08	338.9860
L-Aspartic acid	1.86	132.0270
Fumaric acid	1.76	115.0000
L-Tyrosine	3.10	182.0000
Inosine 5′-monophosphate	7.36	347.0393
Phosphatidylglycerol (16:0/18:1)	13.32	747.5179
Phosphatidylinositol (18:0/18:2)	11.64	835.5000
3-Phosphoglyceric acid	5.12	184.9858
Ascorbic acid	3.13	175.0243
Coumaric acid	3.35	163.0401
Arachidonic acid	13.96	303.2324
Guanosine 5′-triphosphate	5.06	521.9831
Cytidine 5′-monophosphate	7.75	322.0440
3-Hydroxybutyric acid	3.08	103.0395
Folic acid	9.70	440.1326
Oxaloacetic acid	4.03	131.0001
Prostaglandin E2	7.01	351.2206
Lauric acid	13.45	199.1698
Linoleic acid	15.47	279.2324
Oleic acid	13.43	281.2481
α-Ketoglutaric acid	4.53	145.0138
Eicosapentaenoic acid	14.37	301.2168
Heparan sulfate (disaccharide)	8.00	416.0271
L-Cysteine	2.90	120.0121
Guanosine 5′-monophosphate	6.75	362.0503
Succinic acid	3.25	117.0193
Chondroitin sulfate (disaccharide)	6.25	458.0742
Pyruvic acid	2.74	87.0082
Adenosine 5′-triphosphate	10.54	505.9881
Adenosine 5′-monophosphate	7.20	346.0553
Phosphoenolpyruvate	1.84	166.9751
Adenosine 5′-diphosphate	7.94	426.0217
Guanosine 5′-diphosphate	4.58	442.0167
Lithocholic acid	10.67	375.2899
Hypotaurine	1.65	108.0120
Glutathione	6.36	306.0764
Cardiolipin (18:2)4	17.21	1447.9698
Isocitric acid	3.86	191.0196
γ-Glutamylcysteine	3.44	249.0610
L-Tryptophan	3.42	203.0830
Myristic acid	16.43	227.2000
Malic acid	1.92	133.0142
Docosahexaenoic acid	14.90	327.2277
L-Methionine	2.52	148.0435
Phosphatidylserine (16:0/18:1)	8.27	760.5135
Gallic acid	5.20	169.0142
Glutathione disulfide	4.90	611.1445

**Table 2:** Top 50 abundant annotated metabolites in goat bone extract under positive ionization mode ([M + H]^+^).

Annotated Metabolite	RT (Min)	Experimental *m*/*z*
Hypoxanthine	3.38	137.0458
Adenine	4.86	136.0618
Nicotinic acid	7.05	124.0393
Prolyl-Hydroxyproline	3.17	229.1190
Phosphocholine	3.68	184.0712
Lysophosphatidylcholine (18:1)	9.59	522.3554
Creatine	2.14	132.0768
Phosphocreatine	2.59	213.0500
L-Methionine	3.77	150.0660
Lysophosphatidylcholine (16:0)	12.48	496.3398
Phosphatidylcholine (18:0/18:2)	12.14	786.6007
Fumaric acid	4.02	117.0231
Carnosine	6.23	227.1139
L-Phenylalanine	4.32	166.0863
Propionyl-L-carnitine	6.06	218.1387
Putrescine	2.94	89.1073
Lactic acid	2.49	91.0395
Citric acid	4.41	193.0343
L-Aspartic acid	3.87	134.0448
Phosphatidylcholine (16:0/18:1)	16.22	760.5901
Spermine	2.41	203.2230
4-Hydroxyproline	2.75	132.0655
Succinic acid	2.52	119.0350
L-Hydroxyproline	3.87	132.0655
L-Asparagine	2.41	133.0608
Nicotinamide	6.15	123.0553
Spermidine	3.87	146.1652
L-Tyrosine	3.71	182.0812
Betaine	2.42	118.0863
Pyridoxine	2.65	170.0866
Lysophosphatidylcholine (18:0)	5.81	524.3711
Glucosamine	2.57	180.0866
Glutathione disulfide	7.04	613.1592
L-Tryptophan	6.57	205.0977
Pantothenic acid	7.55	220.1239
L-Leucine	4.95	132.1019
D-Galactose	3.39	181.0736
D-Ribose	3.78	151.0601
Hypotaurine	0.99	110.0270
Choline	2.13	104.1070
L-Serine	3.04	106.0499
N-Acetylglucosamine	3.30	222.0977
L-Proline	3.69	116.0706
Riboflavin	10.37	377.1456
Creatinine	3.51	114.0662
Glycyl-Proline	2.01	173.0921
Guanine	3.80	152.0567
Inosine	7.69	269.0880
Xanthine	4.20	153.0407
L-Lysine	3.06	147.1128

## Data Availability

The original contributions presented in this study are included in the article. Further inquiries can be directed to the corresponding authors.

## References

[1] Alqarni L.S., Alghamdi M.D., Alshahrani A.A., Nassar A.M. (2022). Green nanotechnology: Recent research on bioresource-based nanoparticle synthesis and applications. J. Chem..

[2] Nassar A.M., Arafa W.A., Ashammari K., Moustafa S.M., Alsirhani A.M., Hasaneen M.F. (2025). A new green catalyst and antimicrobial agent derived from eco-friendly products of camel bones: Synthesis and physicochemical characterization. Biomass Convers. Biorefinery.

[3] Alanazi A.H., Arafa W.A., Moustafa S.M., Alsohaimi I.H., Elnasr T.A.S., Halawani R.F., Al Zbedy A.S., Nassar A.M. (2025). Green extraction of biomass from waste goat bones for applications in catalysis, wastewater treatment, and water disinfection. J. Hazard. Mater. Adv..

[4] Artamonov M.Y., Sokov E.L., Kornilova L.E., Minenko I.A. (2025). The Intraosseous Environment: Physiological Parameters, Regulatory Mechanisms, and Emerging Insights in Bone Biology. Int. J. Mol. Sci..

[5] Ren M., Wang Y., Yuan Y., Du H., Liang Q., Qin F., Xiong Z. (2025). Integration of UHPLC-MS and mass spectrometry imaging techniques revealed the protective mechanism of Gushudan in postmenopausal osteoporosis rats via branched-chain amino acid metabolism based on the ‘kidney-bone’axis. J. Chromatogr. B.

[6] Jia W., Li R., Wu X., Liu S., Shi L. (2021). UHPLC-Q-Orbitrap HRMS-based quantitative lipidomics reveals the chemical changes of phospholipids during thermal processing methods of Tan sheep meat. Food Chem..

[7] Kret P., Bodzon-Kulakowska A., Drabik A., Ner-Kluza J., Suder P., Smoluch M. (2023). Mass spectrometry imaging of biomaterials. Materials.

[8] Ventura G., Calvano C.D., Bianco M., Castellaneta A., Losito I., Cataldi T.R. (2023). PE, or not PE, that is the question: The case of overlooked lyso-N-acylphosphatidylethanolamines. Rapid Commun. Mass Spectrom..

[9] Dewi D.C., Mahdi C., Sulistyarti H., Aulaniam A. (2023). Identification of volatile compounds in several meat and bone broth using Solid Phase Micro Extraction-Gas Chromatography Mass Spectrometry (SPME-GCMS) for initial detection of Halal and Non-Halal Food. J. Pure Appl. Chem. Res..

[10] Ge X., Gao J., Zhang X., Wang L., Wang C., Fu Z., Yu H., Liu E., Wang L., Liu P. (2024). Comparison of metabolic differences between two geometrical isomers based on multi-omics strategy. Sep. Sci. Plus.

[11] Banerjee I., Mishra D., Das T., Maiti S., Maiti T.K. (2012). Caprine (Goat) collagen: A potential biomaterial for skin tissue engineering. J. Biomater. Sci. Polym. Ed..

[12] Szwed-Georgiou A., Płociński P., Kupikowska-Stobba B., Urbaniak M.M., Rusek-Wala P., Szustakiewicz K., Piszko P., Krupa A., Biernat M., Gazińska M. (2023). Bioactive materials for bone regeneration: Biomolecules and delivery systems. ACS Biomater. Sci. Eng..

[13] León-López A., Fuentes-Jiménez L., Hernández-Fuentes A.D., Campos-Montiel R.G., Aguirre-Álvarez G. (2019). Hydrolysed collagen from sheepskins as a source of functional peptides with antioxidant activity. Int. J. Mol. Sci..

[14] Elias R.J., Kellerby S.S., Decker E.A. (2008). Antioxidant activity of proteins and peptides. Crit. Rev. Food Sci. Nutr..

[15] Fu Y., Therkildsen M., Aluko R.E., Lametsch R. (2019). Exploration of collagen recovered from animal by-products as a precursor of bioactive peptides: Successes and challenges. Crit. Rev. Food Sci. Nutr..

[16] Ideia P., Pinto J., Ferreira R., Figueiredo L., Spínola V., Castilho P.C. (2020). Fish processing industry residues: A review of valuable products extraction and characterization methods. Waste Biomass Valorization.

[17] Alibekov R.S., Alibekova Z.I., Bakhtybekova A.R., Taip F.S., Urazbayeva K.A., Kobzhasarova Z.I. (2024). Review of the slaughter wastes and the meat by-products recycling opportunities. Front. Sustain. Food Syst..

[18] Lin Y.-J., Le G.-W., Wang J.-Y., Li Y.-X., Shi Y.-H., Sun J. (2010). Antioxidative peptides derived from enzyme hydrolysis of bone collagen after microwave assisted acid pre-treatment and nitrogen protection. Int. J. Mol. Sci..

[19] Bonicelli A., Taylor G., Procopio N. (2024). Extraction and untargeted analysis of metabolome from undemineralised cortical bone matrix. Mol. Omics.

[20] Harlina P.W., Maritha V., Musfiroh I., Huda S., Sukri N., Muchtaridi M. (2022). Possibilities of liquid chromatography mass spectrometry (LC-MS)-based metabolomics and lipidomics in the authentication of meat products: A mini review. Korean J. Food Sci. Anim. Resour..

[21] Zhong P., Wei X., Li X., Wei X., Wu S., Huang W., Koidis A., Xu Z., Lei H. (2022). Untargeted metabolomics by liquid chromatography-mass spectrometry for food authentication: A review. Compr. Rev. Food Sci. Food Saf..

[22] Cuykx M., Negreira N., Beirnaert C., Van den Eede N., Rodrigues R., Vanhaecke T., Laukens K., Covaci A. (2017). Tailored liquid chromatography–mass spectrometry analysis improves the coverage of the intracellular metabolome of HepaRG cells. J. Chromatogr. A.

[23] Peris-Vicente J., Esteve-Romero J., Carda-Broch S. (2015). Validation of analytical methods based on chromatographic techniques: An overview. Anal. Sep. Sci..

[24] Marson B.M., Concentino V., Junkert A.M., Fachi M.M., Vilhena R.O., Pontarolo R. (2020). Validation of analytical methods in a pharmaceutical quality system: An overview focused on HPLC methods. Quím. Nova.

[25] Li C., Ozturk-Kerimoglu B., He L., Zhang M., Pan J., Liu Y., Zhang Y., Huang S., Wu Y., Jin G. (2022). Advanced lipidomics in the modern meat industry: Quality traceability, processing requirement, and health concerns. Front. Nutr..

[26] Malinowska J.M., Palosaari T., Sund J., Carpi D., Lloyd G.R., Weber R.J., Whelan M., Viant M.R. (2022). Automated sample preparation and data collection workflow for high-throughput In Vitro metabolomics. Metabolites.

[27] Bispo D.S., Jesus C.S., Correia M., Ferreira F., Bonifazio G., Goodfellow B.J., Oliveira M.B., Mano J.F., Gil A.M. (2022). NMR metabolomics assessment of osteogenic differentiation of adipose-tissue-derived mesenchymal stem cells. J. Proteome Res..

[28] Dhafer C.E.B., Nassar A.M., Alanazi A.H., Alotaibi N.F., Ali H.M., Hasaneen M.F., Moustafa S.M. (2025). Synthesis, structure, thermal, and optical analysis of Ag@ MgO/ZnO: A promising nanocomposite for antimicrobial and antioxidant applications. ChemistrySelect.

[29] Alanazi A.H., Atta A., Bilel H., Halawani R.F., Aloufi F.A., Al Zbedy A.S., Nassar A.M. (2025). Biogenic fabrication of Ag-NPs@ hydroxyapatite from goat bone waste: A sustainable route for photocatalytic and antioxidant applications. Inorganics.

[30] Hering A., Szafrański K., Mikolaszek B., Milewska K., Kucher M., Komala A., Kowalewska A., Stefanowicz-Hajduk J. (2026). Antielastase and Antihyaluronidase Activity of Isomangiferin, a Xanthone with Strong Antioxidant Properties. Int. J. Mol. Sci..

[31] Alanazi A.H., Al Zbedy A.S., Atta A., Moustafa S.M., Ahmed S.H., Alotaibi N.F., Taher I.A., Halawani R.F., Nassar A.M. (2026). Multifunctional Biogenic Silver/Hydroxyapatite Nanocomposite: Photocatalytic Crystal Violet Removal, Antihemolytic Performance, and Broad-Spectrum Antimicrobial Activity. Catalysts.

[32] Al Hajj W., Salla M., Krayem M., Khaled S., Hassan H.F., El Khatib S. (2024). Hydrolyzed collagen: Exploring its applications in the food and beverage industries and assessing its impact on human health–A comprehensive review. Heliyon.

[33] Charlton N.C., Mastyugin M., Török B., Török M. (2023). Structural features of small molecule antioxidants and strategic modifications to improve potential bioactivity. Molecules.

[34] Muhammad S., Latif A., Alam A., Ali M., Elhenawy A.A., Ahmad M., Ahmad S., Ali Shah S.A., Imran S., Ali Khan F. (2026). Synthesis of polyhydroquinoline derivatives as potent antidiabetic and antioxidant agents: In Vitro biological activities and computational studies. Future Med. Chem..

[35] Karakullukcu A.B., Taban E., Ojo O.O. (2023). Biocompatibility of biomaterials and test methods: A review. Mater. Test..

[36] Dwivedi A., Mazumder A., Nasongkla N. (2019). In Vitro and In Vivo biocompatibility of orthopedic bone plate nano-coated with vancomycin loaded niosomes. J. Drug Deliv. Sci. Technol..

[37] Ramadani S.P., Effendi A.N. (2026). Antimicrobial Performance and Hemolytic Compatibility of Medicinal Plant Extracts: A Systematic Review. Int. Health Sci. J..

[38] Mrugesh T., Dipa L., Manishika G. (2011). Effect of lead on human erythrocytes: An In Vitro study. Acta Pol. Pharm..

[39] Lai J.S., Wu T.N., Liou S.H., Shen C.Y., Guu C.F., Ko K.N., Hsueh-Yun C., Chang P.Y. (1997). A study of the relationship between ambient lead and blood lead among lead battery workers. Int. Arch. Occup. Environ. Health.

[40] Bhattacharya S., Gupta N., Dutta A., Bonomo M.G., Milella L., Sarker S.D., Nahar L. (2025). Mitigating ROS signalling pathway-mediated defence mechanism: A novel approach to counteract bacterial resistance using natural antioxidant-based antibiotics. Phytochem. Rev..

[41] Newsholme P., Rebelato E., Abdulkader F., Krause M., Carpinelli A., Curi R. (2012). Reactive oxygen and nitrogen species generation, antioxidant defenses, and β-cell function: A critical role for amino acids. J. Endocrinol..

[42] Marques F., Domingues M.R., Cerqueira M.Â. (2026). Lipid-Based Nanosystems to Enhance the Bioaccessibility and Bioavailability of Algal Lipids. Mol. Nutr. Food Res..

[43] Prasad S., Kashyap R.S., Deopujari J.Y., Purohit H.J., Taori G.M., Daginawala H.F. (2006). Development of an In Vitro method to study clot lysis activity of thrombolytic drugs. Thromb. J..

[44] Amala S., Raghavamma S.T.V., KIranmayi A.H. (2026). Herbal Anticoagulants, Antiplatelets, and Thrombolytics for Cardiovascular Health. Phytomedicine in Cardiovascular Health: Integrating Tradition with Modern Drug Delivery.

[45] Furlan F., Galbiati C., Jorgensen N.R., Jensen J.B., Mrak E., Rubinacci A., Talotta F., Verde P., Blasi F. (2007). Urokinase plasminogen activator receptor affects bone homeostasis by regulating osteoblast and osteoclast function. J. Bone Miner. Res..

[46] Chikha S.B., Bougatef H., Capitani F., Ben Amor I., Maccari F., Gargouri J., Sila A., Volpi N., Bougatef A. (2023). Composition and anticoagulant potential of chondroitin sulfate and dermatan sulfate from inedible parts of garfish (*Belone belone*). Foods.

[47] Cai C., Liu Y., Xu Y., Zhang J., Wei B., Xu C., Wang H. (2025). Mineral-element-chelating activity of food-derived peptides: Influencing factors and enhancement strategies. Crit. Rev. Food Sci. Nutr..

[48] Sripadung P., Rajchakom C., Nunthaboot N., Jiang X., Sungthong B. (2025). Computational and experimental insights into tyrosinase and antioxidant activities of resveratrol and its derivatives: Molecular docking, molecular dynamics simulation, DFT calculation, and In Vitro evaluation. Int. J. Mol. Sci..

[49] Janeczko M., Kochanowicz E. (2026). Hemolytic Activity of Vaginal Candida albicans Isolates and Antifungal Effects of Quinalizarin with Hemolysis Modulation. Pathogens.

[50] Natorska J., Kopytek M., Gołąb A., Waśniowska A., Konieczyńska M., Undas A., Ząbczyk M. (2026). Comparative analysis of whole blood clot viscoelastic properties via thromboelastography and plasma fibrin clot characteristics. Thromb. Res..

[51] Oyaizu M. (1986). Studies on the product of browning reaction prepared from glucosamine. Jpn. J. Nutr..

[52] Chegu K., Mounika K., Rajeswari M., Vanibala N., Sujatha P., Sridurga P., Reddy D.B. (2018). In Vitro study of the anticoagulant activity of some plant extracts. World J. Pharm. Pharm. Sci..

